# De Garengeot Hernia with Acute Gangrenous Appendicitis Case Report

**DOI:** 10.5811/cpcem.35386

**Published:** 2025-03-20

**Authors:** Leon Quach, Alexsandra Biel, Brett Todd

**Affiliations:** * Corewell Health William Beaumont University Hospital, Department of Emergency Medicine, Royal Oak, Michigan; † Oakland University William Beaumont School of Medicine, Rochester, Michigan

**Keywords:** De Garengeot hernia, Amyand hernia, acute gangrenous appendicitis, case report

## Abstract

**Introduction:**

A De Garengeot hernia is defined as a femoral hernia that contains the vermiform appendix. While femoral hernias carrying the appendix are uncommon, strangulation of the appendix in the hernial sac with concurrent acute appendicitis is an extremely rare and life-threatening condition often presenting with an atypical clinical picture.

**Case Report:**

A 51-year-old man presented to the emergency department with two weeks of persistent right inguinal pain after heavy lifting. Imaging revealed suspicion for an Amyand hernia, an inguinal hernia containing a portion of the appendix. However, intraoperative findings revealed a strangulated De Garengeot hernia with gangrenous appendicitis.

**Conclusion:**

De Garengeot hernias are femoral hernias containing the appendix. They are diagnostically challenging and require urgent surgical evaluation and intervention given high risk for strangulation.

## INTRODUCTION

Femoral hernias are protrusions of bowel or abdominal tissue across a weakened abdominal wall into the femoral canal, which is bordered by the inguinal ligament anterosuperiorly, the femoral vein laterally, and the lacunar ligament medially. While femoral hernias make up approximately 5% of all hernias and are not as common as inguinal hernias, 15–20% of all femoral hernias strangulate.^1^ Additionally, an estimated 0.5–5% of femoral hernias contain the appendix, specifically referred to as a De Garengeot hernia.^2^,^3^ Of note, 0.08–1.013% of De Garengeot hernias are complicated by appendicitis.^2^,^3^ Although the combination of physical exam and computed tomography (CT) have proved valuable in assisting with the diagnosis of De Garengeot hernias, the rate of misdiagnosis remains high.^4^ Preoperative diagnosis of De Garengeot hernia is only made in approximately one-third of patients, thus posing diagnostic challenges to the emergency physician.

## CASE REPORT

A 51-year-old male presented to the emergency department (ED) with two weeks of sudden onset, persistent right inguinal pain after heavy lifting. The patient noted that he experienced a constant dull ache with intermittent sharp pain in the right inguinal region and right lower abdominal quadrant, emphasizing that the symptoms seemed similar in nature to his history of kidney stones. He also noticed a bulge in the right inguinal area, which had developed overlying erythema. He had a recent urgent care visit and was prescribed tamsulosin for a presumed kidney stone with no imaging or laboratory studies obtained at the time. The tamsulosin minimally helped with resolution of symptoms. In addition to the inguinal and abdominal pain, he also complained of subjective fevers and chills, intermittent nausea, multiple episodes of non-bloody, non-bilious vomit, and dysuria. Review of systems was otherwise negative for headache, chest pain, shortness of breath, hematochezia, melena, and testicular pain.

Past medical history was significant for multiple sclerosis, attention-deficit/hyperactivity disorder, hypertension, gastroesophageal reflux disease, and chronic lower back pain. Surgical history was notable for laparoscopic gastric fundoplication approximately 10 years prior and tibial open reduction and internal fixation. The patient used marijuana daily. Outpatient medications included amphetamine/dextroamphetamine, cyclobenzaprine, famotidine, hydrocodone and acetaminophen, and tamsulosin.

**Image 1:**
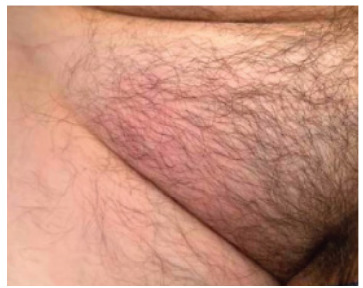
Right groin with overlying erythema and swelling.

Upon arrival, the patient had a heart rate of 103 beats per minute, blood pressure of 134/83 millimeters of mercury, respiratory rate of 22 breaths per minute, oxygen saturation of 99% on room air, and temperature of 36.4 °Celsius. The head, eyes, ear, nose and throat exam was unremarkable. Cardiovascular exam was normal, and pulmonary exam revealed clear and equal breath sounds bilaterally. Abdominal exam was nondistended but notable for a firm abdomen with a tender, nonreducible area of swelling in the right inguinal fold with no guarding or rebound tenderness. Skin exam exhibited overlying erythema in the right inguinal region (Image 1).

**Image 2:**
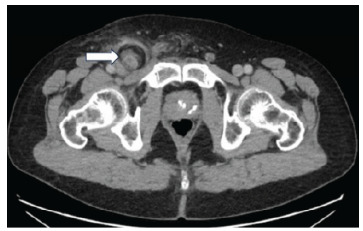
Axial view of computed tomography of the abdomen pelvis showing the appendix within a femoral hernia (arrow).

**Image 3:**
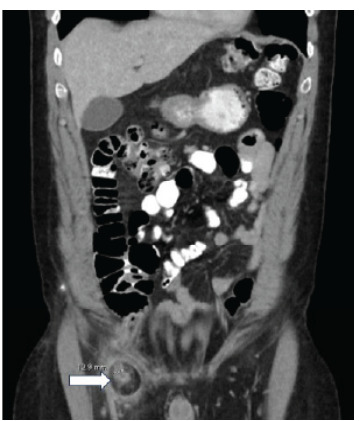
Coronal view of a computed tomography of the abdomen pelvis with a dilated appendix at 12.9 millimeters (arrow) and inflammatory changes around the appendix and hernia.

Initial laboratory studies obtained included a complete blood count, comprehensive metabolic panel, and urinalysis. Complete blood count revealed normal white blood cell count, hemoglobin, and platelet count. Comprehensive metabolic panel showed normal electrolytes and creatinine, and no anion gap. Furthermore, albumin, alkaline phosphatase, aspartate aminotransferase, alanine aminotransferase, and total bilirubin were all within normal limits. Urinalysis showed some ketones and trace leukocyte esterase. Abdominal and pelvis CT with intravenous and oral contrast showed the appendix extending into a fat-containing right groin hernia with evidence of thickening of the appendix and periappendiceal inflammatory change, as well as inflammatory changes around the hernial sac (Images 2, 3).

**CPC-EM Capsule**

What do we already know about this clinical entity?

*While femoral hernias make up approximately 5% of all hernias and are not as common as inguinal hernias, 15*–*20% of all femoral hernias strangulate*.

What makes this presentation of disease reportable?

*We present a rare case of femoral hernia and a unique case of a De Garengeot hernia with the presence of acute gangrenous appendicitis*.

What is the major learning point?

*Given the diagnostic challenges, clinicians should be aware that gangrenous appendicitis is an uncommon, life-threatening complication of the femoral hernia*.

How might this improve emergency medicine practice?

*Early surgical consultation is imperative, as preoperative diagnosis of a De Garengeot hernia is associated with improved patient outcome*.

Appendiceal diameter was noted to be 1.3 centimeters. Other incidental findings included hepatic cysts, a right renal cyst, and diverticulosis. The radiologic diagnosis was an inguinal hernia containing the appendix (Amyand hernia) with concurrent presence of acute appendicitis.

The patient was started on piperacillin-tazobactam. General surgery was consulted and urgently took the patient to the operating room (OR) with a plan for a laparoscopic appendectomy with possible incarcerated right inguinal hernia repair. Intraoperative findings instead showed a femoral hernia with a strangulated gangrenous appendix, known as a De Garengeot hernia. He underwent a successful laparoscopic appendectomy with primary repair of the right femoral hernia. His postoperative course was uncomplicated with the patient tolerating diet and voiding and ambulating independently. He was deemed medically stable and ultimately discharged on postoperative day two.

## DISCUSSION

While lifetime occurrence of a groin hernia is 27–43% in men and 3–6% in women, femoral hernias typically have a female-to-male ratio of 10:1.^1^ Femoral hernias make up only approximately 5% of all groin hernias with the incidence of De Garengeot hernias being less than 5% of all femoral hernias. Consequently, incarceration and strangulation of the appendix within a De Garengeot hernia with concurrent acute appendicitis is an exceedingly rare complication.^2^,^3^ Here we not only present an uncommon case of femoral hernia in a male patient but a unique case of a De Garengeot hernia with the presence of acute gangrenous appendicitis. While De Garengeot hernias have been noted in previous literature, very few cases have documented radiographic and postoperative inconsistencies in diagnosis of this hernia.

Based on CT and physical exam, an Amyand hernia, a protrusion of the appendix into an inguinal rather than a femoral hernia, was the initial clinical suspicion.^5^ Amyand hernias are more common than De Garengeot hernias, representing 0.4–1% of all inguinal hernias with 0.1% of cases being complicated by acute appendicitis.^6^,^7^ Although both Amyand and De Garengeot hernias may present as abdominal protrusions with erythema in the inguinal area, this largely remains a non-specific differentiating finding. Even with the incorporation of modern imaging techniques, CT has only been shown to be diagnostic of De Garengeot hernia 44% of the time.^4^ Given the radiographic challenges in distinguishing inguinal from femoral hernias, preoperative diagnoses of De Garengeot hernias poses diagnostic challenges and uncertainty to the emergency physician where final diagnosis is often only made by chance in the OR.^4^

Although the clinical presentation of a De Garengeot hernia can be fairly atypical and misdiagnosed as an inguinal hernia, high clinical suspicion of a De Garengeot hernia, especially one with concomitant appendicitis, should prompt urgent surgical consultation and evaluation, as the narrow neck of the femoral canal predisposes femoral hernias to high risk of strangulation and further complications as compared to inguinal hernias.^4^,^8^ De Garengeot hernias should not be reduced in the ED due to the complex nature of the hernia and its risk for appendicitis.^4^ Emergency physicians should be aware that if left untreated, the inflammation may progress to life-threatening complications, which include necrosis of the hernia contents, bowel obstruction, necrotizing fasciitis, and abscess formation.^4^ Imaging studies can aid in the diagnosis and help guide clinical decision-making, particularly when considering the possibility of incarceration, strangulation, or concurrent acute appendicitis. However, clinical presentation is non-specific, and precise knowledge of hernia sac content preoperatively is not mandatory when considering surgical intervention.^8^

As evidenced in this case, the De Garengeot hernia with gangrenous appendicitis was not made until the patient was in the OR where he underwent laparoscopic appendectomy and open, right femoral hernia repair. Notably, preoperative diagnosis of a De Garengeot hernia is associated with a lower complication rate and shorter hospital length of stay than intraoperative diagnosis.^4^ Given the lack of efficacy in radiographic methods for diagnosing a De Garengeot hernia, particularly with CT being less than 50% diagnostic, prompt surgical exploration should be pursued if there is high clinical suspicion for an incarcerated or strangulated femoral hernia with abdominal contents.

## CONCLUSION

A De Garengeot hernia is defined as a femoral hernia that contains the vermiform appendix. Given its diagnostic challenges, emergency physicians should know that acute gangrenous appendicitis is an uncommon and life-threatening complication of this femoral hernia.
